# Nanostructured Hydrogels by Blend Electrospinning of Polycaprolactone/Gelatin Nanofibers

**DOI:** 10.3390/nano8070551

**Published:** 2018-07-20

**Authors:** Lode Daelemans, Iline Steyaert, Ella Schoolaert, Camille Goudenhooft, Hubert Rahier, Karen De Clerck

**Affiliations:** 1 Department of Materials, Textiles and Chemical Engineering (MaTCh), Ghent University, Technologiepark 907, 9052 Ghent, Belgium; lode.daelemans@ugent.be (L.D.); iline.steyaert@ugent.be (I.S.); ella.schoolaert@ugent.be (E.S.); camille.goudenhooft@univ-ubs.fr (C.G.); 2 Research Unit of Physical Chemistry and Polymer Science, Department of Materials and Chemistry, Vrije Universiteit Brussel, Pleinlaan 2, 1050 Brussels, Belgium; hrahier@vub.ac.be

**Keywords:** biomaterial, biomedical, nanofibers, scaffolds, reinforced, hybrid material, thermal analysis, nanofibrous membranes

## Abstract

Nanofibrous membranes based on polycaprolactone (PCL) have a large potential for use in biomedical applications but are limited by the hydrophobicity of PCL. Blend electrospinning of PCL with other biomedical suited materials, such as gelatin (Gt) allows for the design of better and new materials. This study investigates the possibility of blend electrospinning PCL/Gt nanofibrous membranes which can be used to design a range of novel materials better suited for biomedical applications. The electrospinnability and stability of PCL/Gt blend nanofibers from a non-toxic acid solvent system are investigated. The solvent system developed in this work allows good electrospinnable emulsions for the whole PCL/Gt composition range. Uniform bead-free nanofibers can easily be produced, and the resulting fiber diameter can be tuned by altering the total polymer concentration. Addition of small amounts of water stabilizes the electrospinning emulsions, allowing the electrospinning of large and homogeneous nanofibrous structures over a prolonged period. The resulting blend nanofibrous membranes are analyzed for their composition, morphology, and homogeneity. Cold-gelling experiments on these novel membranes show the possibility of obtaining water-stable PCL/Gt nanofibrous membranes, as well as nanostructured hydrogels reinforced with nanofibers. Both material classes provide a high potential for designing new material applications.

## 1. Introduction

Electrospun nanofiber membranes have gained a lot of attention the past few years. Their striking resemblance to the morphology of the extracellular matrix (ECM) made them especially interesting for biomedical applications. Nanofibrous membranes can, unlike structures with porosity on a larger scale, provide an environment closely mimicking the natural ECM by providing appropriate cell binding sites. Cell behavior and functionality can be controlled by man-made nanofibrous scaffolds, which are therefore increasingly being used in tissue engineering and wound-healing applications [1,2]. Solvent electrospinning provides a relatively simple way to produce nanofibers from polymer solutions and is therefore currently the most commonly applied processing technique. To fully exploit the potential of electrospun nanofibrous materials, further research toward the use of less toxic, more economical solvents and a stable electrospinning process suitable for upscaling, is essential.

Polycaprolactone (PCL) is commonly used in biomedical products, ranging from sutures or staples for wound closure to contraceptive devices. This is mainly due to its tunable degradation profile, in combination with low cost, easy production and availability of medical-grade material [3,4]. It is thus not surprising that PCL nanofibers for biomedical applications have also been developed [5,6,7,8,9,10]. The practical application of PCL nanofibers in the biomedical field, however, is severely limited due to the hydrophobic properties adversely affecting cell affinity. Although the nanofiber morphology closely resembles the ECM, its complexity in terms of composition, biological functionality and biophysical properties makes it difficult to reproduce using single polymer systems. Blending synthetic and natural polymers offers the opportunity to tune physical properties and bioactivity while minimizing the disadvantages of both polymer components, thus more closely resembling the native ECM [2,11,12,13]. Compared to the use of copolymers or modified nanofibers, blending offers an alternative approach that is simple and economical [14].

A wide variety of natural polymers are available for biomedical applications, including several polypeptides and polysaccharides. They provide the desired biological properties in a blend and are biodegradable. Gelatin (Gt) is a commercial polypeptide widely available in medical-grade material and very suitable for biomimetic applications since it displays many integrin binding sites for cell adhesion and differentiation [15,16,17,18,19]. Although it is possible to produce pure Gt nanofibers [20], blending with PCL can give rise to superior mechanical properties [21,22,23], increasing its application potential.

Our previous work showed that Gt nanofibers are cold-water-soluble due to their high surface-to-volume ratio which promotes water penetration and dissolution. They can be used as an instant cold gel. This presents opportunities for PCL/Gt blended nanofibrous membranes to design new hybrid materials for biomedical applications. In this article, we develop a non-toxic and economical solvent system for the production of PCL/Gt blend nanofibers by electrospinning. An acetic acid (AA)/formic acid (FA) solvent system is selected based on our previous work in which pure PCL and pure Gt nanofibers were produced [5,20]. Several PCL/Gt blend nanofibrous membranes with varying compositions are electrospun and their morphology is characterized. Cold-gelling experiments are performed on nanofibrous membranes with varying PCL/Gt ratio to study its effect on the gel formation of the Gt component and the resulting structure of these novel materials.

## 2. Materials and Methods

### 2.1. Materials

Polycaprolactone (average M_n_ of 80 kg·mol^−1^) was purchased from Sigma-Aldrich (Overijse, Belgium). Commercial gelatin isolated from pigskin by the acidic process (type A pharma-grade, 300 Bloom) was kindly supplied by Rousselot, Ghent, Belgium. Both polymers were used as-received for the electrospinning process. AA (99.8 vol%) and FA (98.0 vol%) were supplied by Sigma-Aldrich (Overijse, Belgium).

Efforts in optimization also included testing of a lower molecular weight PCL (average M_w_ 14 kg·mol^−1^) purchased from Sigma-Aldrich (Overijse, Belgium) and a gelatin isolated from bovine bones by the alkaline process (type B pharma-grade, 260 Bloom) kindly supplied by Rousselot, Ghent, Belgium.

### 2.2. Electrospinning

The electrospinning process allows for easy production of nanofibers using polymer solutions, and is more thoroughly described for PCL and gelatin in our previous papers [5,20]. Blend nanofibers are obtained when the solution used for electrospinning contains two (or more) polymer components.

The PCL/Gt electrospinning solutions were obtained by simultaneous dissolution of PCL and gelatin, allowing for variations in solvent ratio, total polymer concentration and polymer ratio. The electrospinning solutions were characterized prior to use by their viscosity and conductivity. Viscosity was measured using a Brookfield viscometer LVDV-II (standard deviation was on average 8%, Brookfield, Middleboro, MA, USA) and conductivity was determined by a CDM210 conductivity meter of Radiometer Analytical (standard deviation was on average 11%, Düsseldorf, Germany).

All electrospinning trials for process optimization were performed using a mono-nozzle setup, leading to a scalable electrospinning process. Nanofibrous membranes for subsequent analysis were electrospun using a multinozzle or rotating drum setup at low speed [24,25], already providing upscaled samples. The latter samples were electrospun using the AA/FA solvent system (solvent ratio as specified in the text), a polymer concentration of 13 wt% (PCL/Gt ratio as specified in the text), a flow rate of 1 mL·h^−1^, a tip-to-collector distance of 12.5 cm and the applied voltage adapted for stable electrospinning.

The electrospun samples were examined by scanning electron microscopy (FEI Quanta 200 F, Thermo Fisher Scientific, Eindhoven, The Netherlands) at an accelerating voltage of 20 kV. Sample preparation was done using a sputter coater (Emitech SC7620, coating with Au, Rotterdam, The Netherlands). The nanofiber diameters were measured using ImageJ version 1.48, https://imagej.net. The average fiber diameters and their standard deviations are based on 50 measurements per sample.

### 2.3. Water Treatment of PCL/Gt Blends

Water treatment of PCL/Gt blend nanofibrous membranes was done by washing the samples in a large quantity of demineralized water at 35 °C (maximal temperature without affecting PCL nanofibrous structure) for 30 s. Four repetitions were done before further drying at ambient temperature for over 24 h.

### 2.4. Materials Characterization

Solution cast films (prepared by solution casting in Teflon evaporation dishes of the electrospinning solution) and nanofibrous membranes were characterized using a Fourier Transform Infrared (FTIR, Thermo Fisher Scientific, Eindhoven, The Netherlands) spectrometer with Attenuated Total Reflectance (ATR) accessory (diamond crystal) from Thermo Scientific. The spectra were recorded in the range 4000–400 cm^−1^ with a resolution of 4 cm^−1^. 32 scans were averaged for each spectrum. The films were prepared by sampling the electrospinning solution and solution casting onto Teflon evaporation dishes. All nanofibrous membranes were measured as-spun. Additionally, PCL/Gt nanofibrous membranes were also measured after (partial) removal of gelatin by water treatment as detailed above.

Differential scanning calorimetry (DSC) measurements were performed using a TA Instruments (Asse, Belgium) Q2000 equipped with a refrigerated cooling system and using nitrogen as purge gas (50 mL·min^−1^). The instrument was calibrated using Tzero technology, including a temperature calibration with indium. DSC measurements were performed on samples of 3.00 ± 0.30 mg conditioned at 65% RH for 24 h, enclosed in hermetic Tzero aluminum crucibles and at a heating rate of 2.5 K·min^−1^. The standard deviation of the melting enthalpy of PCL nanofibers was 5%.

Dynamic Mechanical Analysis (DMA) measurements were done using a TA Instruments Q800 (Asse, Belgium), equipped with liquid nitrogen cooling (LNCS) and film tension clamps. Calibration was done according to a manufacture-defined procedure. Temperature calibration was performed by means of the melting transition of gallium. Films and nanofibrous membranes were measured in film tension mode, using a Poisson’s ratio of 0.44, a frequency of 1 Hz, a strain of 0.5%, a static force of 0.01 N and a heating rate of 2.5 K·min^−1^. The measurements were performed on as-spun nanofibrous membranes at room humidity (40 ± 10% RH). The samples were not heated above 30 °C to avoid melting of the PCL component.

## 3. Results and Discussion

### 3.1. Electrospinning of PCL/Gt Blend Nnanofibers Using AA/FA

Our previous investigation of the pure components illustrated that both PCL and gelatin are electrospinnable when dissolved in AA/FA, and this for several concentrations and solvent ratios [5,20]. There is a significant overlap of solution and processing parameters for the stable electrospinning of the pure polymers. Based hereon, a total polymer concentration of 13 wt% and a 70/30 AA/FA solvent system were chosen as a starting point for the blend electrospinning (tip-to-collector distance (TCD)) of 12.5 mm, flow rate of 1 mL·h^−1^. This solvent choice minimizes the amount of FA, which minimizes degradation of PCL [5], while guaranteeing electrospinning of fine bead-free PCL and gelatin nanofibers.

**Figure 1:**
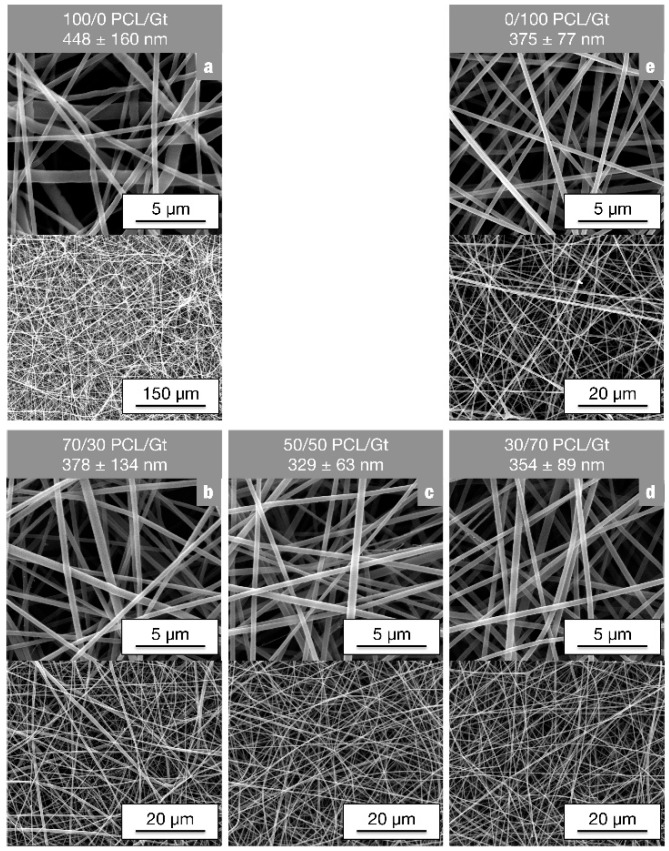
Scanning electron microscopy (SEM) images of PCL/Gt blend nanofibers electrospun using a total polymer concentration of 13 wt% and 70/30 AA/FA (TCD of 12.5 cm, flow rate of 1 mL·h^−1^ and voltage adjusted in the range of 15–20 kV for stable electrospinning): (**a**) 100/0 PCL/Gt, (**b**) 70/30 PCL/Gt, (**c**) 50/50 PCL/Gt, (**d**) 30/70 PCL/Gt and (**e**) 0/100 PCL/Gt. All PCL/Gt blend ratios were well electrospinnable.

**Figure 2:**
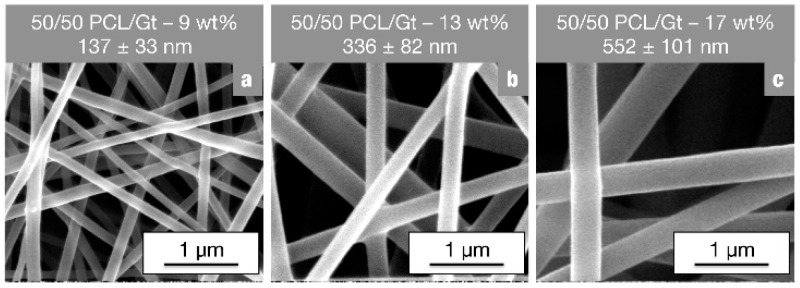
SEM images of 50/50 PCL/Gt blend nanofibers electrospun using 70/30 AA/FA (TCD of 12.5 cm, FR of 1 mL·h^−1^ and E adjusted for stable electrospinning) with a total polymer concentration of (**a**) 9 wt%, (**b**) 13 wt% and (**c**) 17 wt%. Varying the total polymer concentration significantly affects fiber diameters.

As illustrated in Figure 1, the full range of PCL/Gt blend ratios is well electrospinnable using the chosen parameters. Indeed, uniform bead-free PCL/Gt nanofibers could be produced in a stable manner for all compositions. Although the conductivity of the electrospinning solution increases significantly, and the viscosity markedly drops with increasing gelatin content (Figure S1), no remarkable differences in nanofiber diameter were observed. Variations in total polymer concentration with a fixed polymer ratio, on the other hand, do affect fiber diameter. Electrospinning of a 50/50 PCL/Gt blend solution results in a stable process and bead-free nanofibers for polymer concentrations between 9 wt% and 17 wt%. Within this electrospinnable window, the nanofiber diameter could be tuned between 140 nm and 550 nm respectively (Figure 2). Blend electrospinning of PCL/Gt using the AA/FA solvent system is thus highly flexible, with both the PCL/Gt ratio and the fiber diameter adjustable to the end application.

Although this clearly did not impede electrospinnability, all the PCL/Gt blend solutions were opaque due to a phase separation resulting in emulsions. The homogeneous emulsions gradually evolved toward two completely separated clear phases over the course of a few hours after complete dissolution. Within this time frame, however, neither process stability nor fiber morphology were affected. Additionally, continued stirring of the emulsions makes stable electrospinning possible for much longer dwell times (no visible phase separation after 48 h).

PCL degrades significantly in the AA/FA solvent system with prolonged dwell times in solution [5]. Therefore, the polymer blend degradation was investigated for longer dwell times in solution through viscosity measurements (Figure S2). Although the reduction in solution viscosity is not as large as for pure PCL (about 40% after 48 h in 70/30 AA/FA), 50/50 PCL/Gt blends are also characterized by a decrease in viscosity with increasing dwell time in the acid solution (about 20% after 48 h in 70/30 AA/FA). This is probably mainly due to PCL degradation [20], resulting in slightly smaller fiber diameters (276 ± 67 nm after 3 h in solution vs. 233 ± 47 nm after 48 h in solution). Despite the small change in fiber morphology, process stability was not affected by PCL degradation. Using a continuously stirred PCL/Gt emulsion in 70/30 AA/FA, uniform bead-free nanofibrous membranes can thus be produced over the course of 48 h, with only a slight influence on fiber diameter.

As the electrospinning remains stable for a prolonged time, PCL/Gt blend electrospinning using the AA/FA solvent system was readily scalable to produce large nanofibrous membranes. Homogeneous nanofibrous membranes with a nominal size of 300 × 300 mm² were produced on a multinozzle setup. Their uniformity was validated by using SEM and FTIR analysis at several points throughout the membrane. No significant differences in fiber morphology or diameter were observed. Similarly, for all PCL/Gt blend ratios, FTIR spectra were identical within a single membrane, even though they were electrospun using emulsions. Pure PCL and pure gelatin are characterized by a few non-overlapping peaks in their FTIR spectra (Figure S3 and Table S1) [22,26,27]. Therefore, a qualitative indication of the PCL/Gt composition of the nanofibrous membranes is given by the ration of the carbonyl stretching peak of PCL and the amide I peak of gelatin. The 70/30 AA/FA solvent system thus allows for stable, reproducible, and scalable production of PCL/Gt blend nanofibers with high flexibility in fiber composition and diameter.

### 3.2. Stabilizing the Electrospinning Emulsions by Tuning the Solvent System

**Figure 3:**
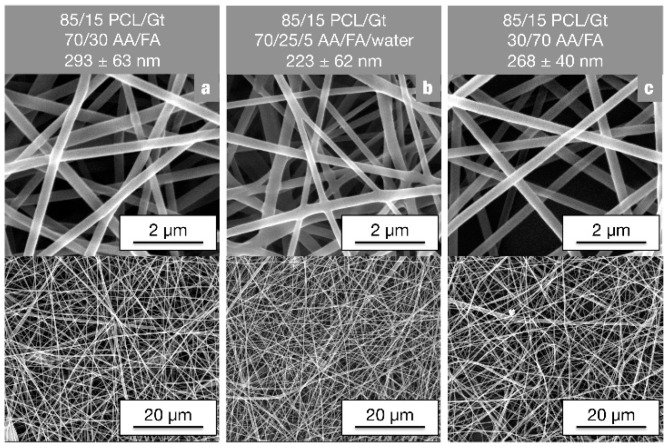
SEM images of 85/15 PCL/Gt nanofibers produced using a polymer concentration of 13 wt% but with differing solvent systems: (**a**) An emulsion unstable in time; (**b**) An emulsion stable in time, and; (**c**) A clear solution stable in time.

Water could be added to the electrospinning solutions up to 5 vol% without affecting solubility of the polymers. It stabilizes the solutions and hinders the phase separation of the PCL/Gt blends into two completely separated phase (the gelatin-rich phase settles at the bottom of the container without continued stirring). This makes electrospinning of bead-free PCL/Gt blend nanofibers possible for low gelatin concentrations without continued stirring (Figure 3b). Moreover, using a 70/25/5 AA/FA/water solvent system, the settling phenomenon can be prevented for gelatin concentrations up to 20 wt% of the total polymer mass. A similar effect has been reported by Feng et al., who illustrated that addition of a small amount of AA to a trifluoroethanol solution increases PCL-gelatin miscibility [22].

**Figure 4:**
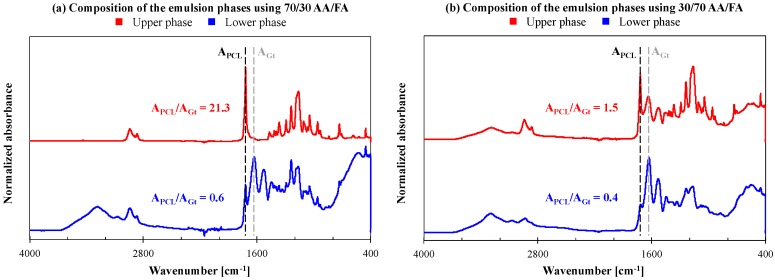
Normalized Attenuated Total Reflectance-Fourier-transform infrared spectroscopy (ATR-FTIR) spectra of films obtained by solution casting the upper (red) or lower (blue) phase, representing the continuous and dispersed phase respectively, of a PCL/Gt emulsion in (**a**) 70/30 AA/FA or (**b**) 30/70 AA/FA left at rest.

Alternatively, the effect of a changing the AA/FA solvent ratio was investigated. With an increasing FA concentration, miscibility is slightly improved. Although the gelatin-rich phase still settles on the bottom of the container when stirring is stopped, the PCL-rich upper phase contains more gelatin with decreasing AA/FA ratio of the solution. This is clearly illustrated by FTIR analysis of solution cast films of the upper and lower phase of an emulsion kept at rest (Figure 4). The gelatin peaks of the PCL-rich upper phase are much more pronounced when dissolved in 30/70 AA/FA (A_PCL_/A_Gt_ = 1.5) than in 70/30 AA/FA (A_PCL_/A_Gt_ = 21.3) (Figure 4 red curves, b vs. a), pointing to a higher gelatin concentration. Due to this higher miscibility in the PCL-rich phase, clear solutions stable in time were obtained for gelatin concentrations up to 30 wt% of the total polymer mass when using the 30/70 AA/FA solvent system. These clear and stable solutions were well electrospinnable, giving rise to bead-free nanofibers with reproducible diameters (Figure 3c). FTIR analysis of the obtained nanofibrous membranes, however, showed no differences in composition compared to the membranes electrospun using emulsions in 70/30 AA/FA (Figure S4). Consequently, the increased miscibility in 30/70 AA/FA does not affect nanofiber composition on the mm-scale.

### 3.3. Interactions between the PCL and Gt Components by Thermal Analysis

The interactions and miscibility of polymer components in a blend often affect the thermal properties of the blend compared to their pure counterparts. Indeed, in a polymer blend containing a crystallizable component, a decrease in melting temperature and/or crystallinity is often observed; interaction between chains and chain segments of the blend components can cause a decrease in lamellar thickness of the crystals and/or a decrease in the amount of crystallizable material [28,29,30,31]. Additional to changes in melting behavior, also the glass transition is possibly affected by blending [28,32], with completely miscible polymer blends showing only one intermediate T_g_. Therefore, the PCL/Gt blend nanofibers were analyzed using DSC and DMA and compared to the melting behavior and glass transition to pure PCL nanofibers.

**Figure 5:**
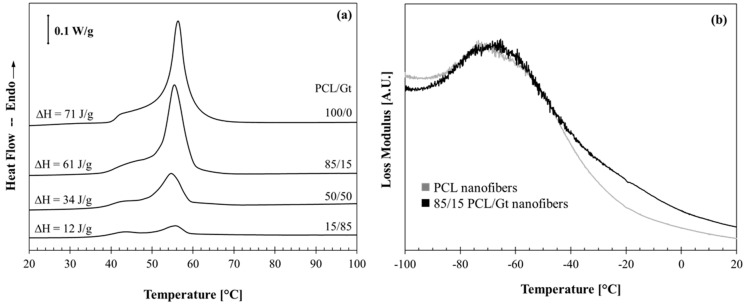
The effect of blending with gelatin, using a 70/30 AA/FA solvent system, on (**a**) the melting behavior and (**b**) the glass transition of PCL-based nanofibers measured by DSC at 2.5 K·min^−1^ and by DMA respectively.

While studying PCL/Gt blend nanofibers using DSC, care has to be taken when analyzing the PCL melting endotherm, since dissociation of gelatin triple helices occurs within the same temperature range [20]. However, the heat effect of this triple helix dissociation is far less than the melting enthalpy measured for pure PCL nanofibers, namely about 4 J·g^−1^ and about 70 J·g^−1^ respectively. The endothermic transition can thus mainly be attributed to the PCL component. As expected, DSC analysis of PCL/Gt blend nanofibers shows a clear decrease in overall melting enthalpy (Figure 5a). This decrease is in line with the decreasing PCL concentration. Indeed, recalculation of the overall melting enthalpy as a function of PCL mass results in values of about 70 J·g^−1^ for all samples (Table 1). The value for 15/85 PCL/Gt nanofibers is slightly higher, probably due to a more significant overlapping heat effect of the triple helix dissociation within the gelatin component and a larger error. Overall, however, the PCL melting enthalpy does not seem to be significantly decreased due to blending. Additionally, the melting trace showed a peak value (T_p_) of 55 ± 1 °C for all samples. These results indicate that blending of PCL with gelatin does not significantly affect the melting behavior of the PCL component and interaction between the components is thus low. This also confirms that the analyzed nanofibers contain the expected amount of PCL and gelatin, pointing to a homogeneous blend composition throughout the membranes. Similar results were obtained for all investigated solvent systems (70/30 AA/FA, 30/70 AA/FA and 70/25/5 AA/FA/water). Nanofiber composition, uniformity and PCL-gelatin interactions are thus not affected when electrospinning a clear solution compared to an emulsion. DMA analysis on 85/15 PCL/Gt blend nanofibers illustrated that the glass transition of PCL is hardly affected compared to pure PCL (Figure 5b). This again points to weak interactions and low miscibility between PCL and gelatin.

### 3.4. Cold-Water Solubility of the Gelatin Component

As discussed in our previous paper, gelatin nanofibers are cold-water-soluble and dissolve to form a hydrogel in water at room temperature when they are not cross-linked [20]. Blending with PCL could affect this property. The 85/15 PCL/Gt nanofibers do not show significant changes in nanofiber morphology when submerged in cold water, suggesting the gelatin component to be not dissolved. With increasing gelatin concentration, however, a fiber-reinforced hydrogel is obtained, where gelatin partly gels but a nanofibrous network mainly consisting of PCL remains. Although water stability through cross-linking can easily be obtained [33], nanofiber-reinforced gelatin hydrogels could prove to be a promising biomedical material [34,35,36], combining a soft and elastic consistency while allowing for cell support on a nanofibrous membrane with increased porosity. To characterize the nanofibrous reinforcing structure and gain insight into the phase morphology of PCL/Gt nanofibers, the water-soluble gelatin fraction was removed using water of 35 °C.

**Figure 6:**
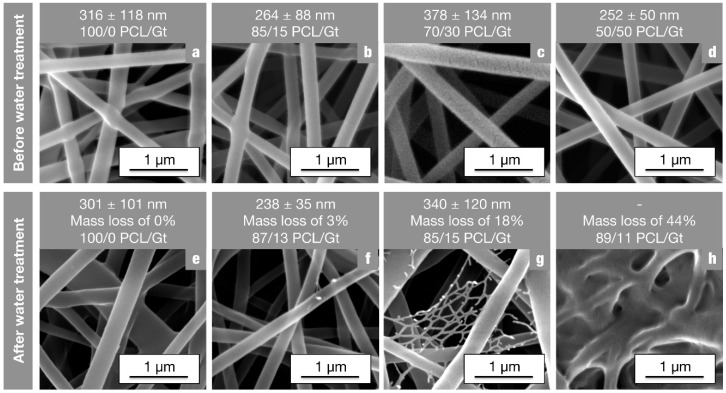
SEM images of PCL/Gt blend nanofibers (13 wt%, 70/30 AA/FA) with different PCL/Gt ratios (**a**–**d**) before and (**e**–**h**) after water treatment, i.e., rinsing with demineralized water at 35 °C. Mass loss is calculated by weighing the dried weight. Based on this mass loss of gelatin, a new PCL/Gt ratio was calculated for the rinsed samples.

All nanofibrous membranes having a PCL concentration of ≥50% still showed structural integrity after water treatment. It was, therefore, possible to characterize them using SEM and FTIR (Figure 6 and Table 2). Additionally, the mass loss of the membranes was determined. Since the water treatment procedure does not affect pure PCL nanofibers (Figure 6a,e), this mass reduction can be attributed to a decrease in gelatin content, resulting in a new PCL/Gt ratio.

SEM analysis, the measured mass loss and FTIR analysis all clearly indicate that the rinsing procedure only has a minor influence on 85/15 PCL/Gt nanofibers. Indeed, fiber morphology before and after water treatment is comparable (Figure 6b,f), and the FTIR spectra were identical, resulting in similar A_PCL_/A_Gt_ ratios (Table 2). A similar result was obtained for 85/15 PCL/Gt nanofibers electrospun using 30/70 AA/FA (clear solutions). The phase separation in the electrospinning solution thus only has a minor effect on the final PCL/Gt fiber morphology. Although PCL-gelatin interaction within the nanofibers is low according to our thermal analysis, the gelatin component no longer dissolves in water. This indicates that the gelatin-rich phase is finely dispersed throughout the nanofibers, so that the hydrophobicity of PCL prevents gelatin dissolution.

With increasing gelatin content, there is a significant amount of gelatin dissolved by rinsing in demineralized water at 35 °C, as evidenced by the mass loss and the decreasing amide I peak of gelatin in the FTIR spectra (Figure 6 and Table 2 respectively). The influence on the fiber diameter; however, is only small, especially for 70/30 PCL/Gt nanofibers, and no porosity of individual nanofibers is observed (Figure 6g). Additionally, the new PCL/Gt ratio after extraction is about 85/15. It is, therefore, hypothesized that a 70/30 PCL/Gt nanofibrous membrane is built up by a mixture of PCL-rich and gelatin-rich nanofibers. The PCL-rich nanofibers within the membrane contain about 15 wt% of gelatin and are not affected by water treatment, similar to 85/15 PCL/Gt nanofibers. The gelatin-rich nanofibers are dissolved, possibly leaving some PCL residue upon rinsing (Figure 6g). Although there are clearly still intact nanofibers present in the 50/50 PCL/Gt nanofibrous membranes after water treatment, a gelatin film is observed in SEM analysis (Figure 6h). This indicates that the gelatin polypeptide chains are sufficiently immobilized by entanglements with the PCL component to prevent removal by dissolution. Moreover, the gelatin phase must be present more toward the shell of the nanofibers, causing film formation and a slightly higher A_PCL_/A_Gt_ ratio in FTIR (Table 2).

These results indicate that PCL/Gt blend nanofibrous membranes have a high potential for advanced biomedical material design. On one hand, water-stable PCL/Gt blend nanofibrous membranes can be produced for gelatin concentrations up to 15 wt%. On the other hand, for higher amounts of gelatin, cold-gelling when in contact with water is possible, giving rise to a nanostructured hydrogel that is physically reinforced by PCL nanofibers. The nanofibers in these nanostructured reinforced hydrogels consist of PCL-rich nanofibers that still contain a significant amount of gelatin (about 10 to 15 wt%).

## 4. Conclusions

In conclusion, the AA/FA-based solvent system allows for stable, reproducible, and scalable PCL/Gt blend electrospinning with high flexibility, this while the toxicity of the solvent system is significantly reduced compared to traditionally utilized solvents. Indeed, dissolution of PCL/Gt blends in 70/30 AA/FA yields well electrospinnable emulsions for the whole PCL/Gt composition range. Although these emulsions are subject to settling of the gelatin-rich phase, uniform bead-free nanofibers can easily be produced within a time frame of a few hours or by continued stirring of the electrospinning solution. Additionally, the resulting fiber diameter can be tuned by altering the total polymer concentration. Using 30/70 AA/FA, stable and clear electrospinning solutions are obtained for gelatin concentrations up to 30 wt% due to increased miscibility. Further augmentation of gelatin concentration results in phase separation with the formation of an unstable emulsion. For all PCL/Gt ratios, process stability is best, and toxicity is minimized using 70/30 AA/FA. For gelatin concentrations up to 20 wt%, substitution of 5 vol% acid with water stabilizes the emulsion for over 48 h, making electrospinning possible without continued stirring or compromising the lower toxicity of the solvent system.

The resulting PCL/Gt nanofibrous membranes are water-stable up to gelatin concentrations of 15 wt%, irrespective of the possible phase separation in the electrospinning solution used for production (emulsion vs. clear miscible solution). It is hypothesized that membranes containing a higher amount of gelatin are built up of a mixture of nanofibers mainly consisting of PCL (~85/15 PCL/Gt) and nanofibers mainly consisting of cold-water-soluble and cold-gelling gelatin. The PCL-gelatin interactions within the nanofibers were low, as blending did not seem to affect the glass transition or melting of the PCL component. If the original nanofibrous membrane contains a high amount of gelatin (>15 wt%), cold-gelling when in contact with water is possible, giving rise to a nanofiber-reinforced physical gelatin hydrogel. This novel combination of material properties could prove to be a promising material for biomedical applications.

## Figures and Tables

**Table 1:** Melting enthalpy measured using DSC (Figure 5a), as a function of total polymer mass and recalculated as a function of PCL mass.

Nanofibrous Membranes	∆H_m_ (Joules per Gram Fiber)	∆H_m_ (Joules per Gram PCL)
PCL	71	71
85/15 PCL/Gt	61	72
50/50 PCL/Gt	29	68
15/85 PCL/Gt	12	80

**Table 2:** Composition of the nanofibrous membranes before and after water treatment, analyzed using the ratio of the carbonyl stretching peak of PCL and the amide I peak of gelatin measured using FTIR. After water treatment, the composition is comparable and reflecting a gelatin concentration of about 15 wt%.

Nanofibrous Membranes	A_PCL_/A_Gt_ before Water Treatment	A_PCL_/A_Gt_ after Water Treatment
85/15 PCL/Gt	4.9	5
70/30 PCL/Gt	2.3	4.7
50/50 PCL/Gt	0.9	3.1 *

* A slightly higher gelatin concentration is measured, probably due to the gelatin film covering the membrane, as demonstrated in Figure 6h.

## Supplementary Materials

The following are available online at http://www.mdpi.com/2079-4991/8/7/551/s1. Figure S1. Viscosity and conductivity measurements of PCL/Gt blend electrospinning solutions, using the 70/30 AA/FA solvent system (a) as a function of the PCL/Gt ratio and (b) as a function of the total polymer concentration. Figure S2. Viscosity measurements of the electrospinning solutions (PCL and/or Gt dissolved in 70/30 AA/FA) with increasing dwell time show that PCL degrades substantially whereas the Gt component remains quite stable. Figure S3. ATR-FTIR spectra of pure PCL and pure Gt, showing their characteristic peaks. Figure S4. Normalized ATR-FTIR spectra of 85/15 PCL/Gt blend nanofibers electrospun using an emulsion (dissolution in 70/30 AA/FA) or a clear solution (dissolution in 30/70 AA/FA). Table S1. Characteristic peaks of a PCL pellet and Gt powder in ATR-FTIR, as indicated in Figure S3.
