# Supplementary material for: Nanostructured Hydrogels by Blend Electrospinning of Polycaprolactone/Gelatin Nanofibers
Source: Nanomaterials (Basel). 2018 Jul 20;8(7):551. doi: 10.3390/nano8070551 (PMC6070828; doi:10.3390/nano8070551)
Supplement: Supplementary file 1 [file nanomaterials-08-00551-s001.pdf]

Nanostructured Hydrogels by Blend Electrospinning of Polycaprolactone/Gelatin Nanofibers

Lode Daelemans [1,†], Iline Steyaert [1,2,†], Ella Schoolaert [1], Camille Goudenhooft [1], Hubert Rahier [2], and Karen De Clerck [1,*]

1. Department of Materials, Textiles and Chemical Engineering (MaTCh), Ghent University, Technologiepark 907, 9052 Ghent, Belgium; lode.daelemans@ugent.be (L.D.); iline.steyaert@ugent.be (I.S.); ella.schoolaert@ugent.be (E.S.); camille.goudenhooft@univ-ubs.fr (C.G.)
2. Research Unit of Physical Chemistry and Polymer Science, Department of Materials and Chemistry, Vrije Universiteit Brussel, Pleinlaan 2, 1050 Brussels, Belgium; iline.steyaert@ugent.be (I.S.); hrahier@vub.ac.be (H.R.)

† These authors contributed equally to this work.

Supporting information

Electrospinning of PCL/Gt nanofibers: solution properties

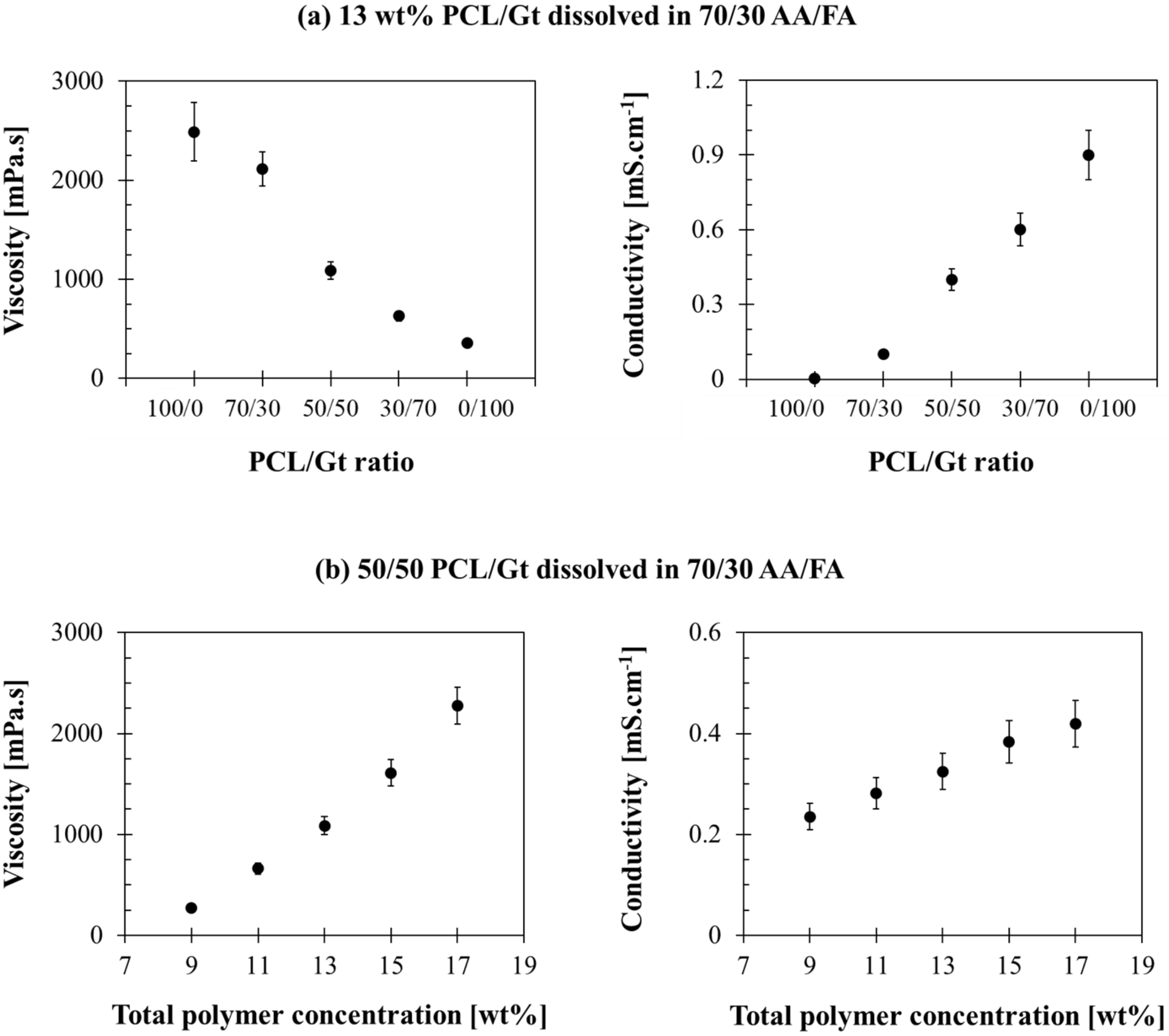

Figure S1. Viscosity and conductivity measurements of PCL/Gt blend electrospinning solutions, using the 70/30 AA/FA solvent system (**a**) as a function of the PCL/Gt ratio and (**b**) as a function of the total polymer concentration.

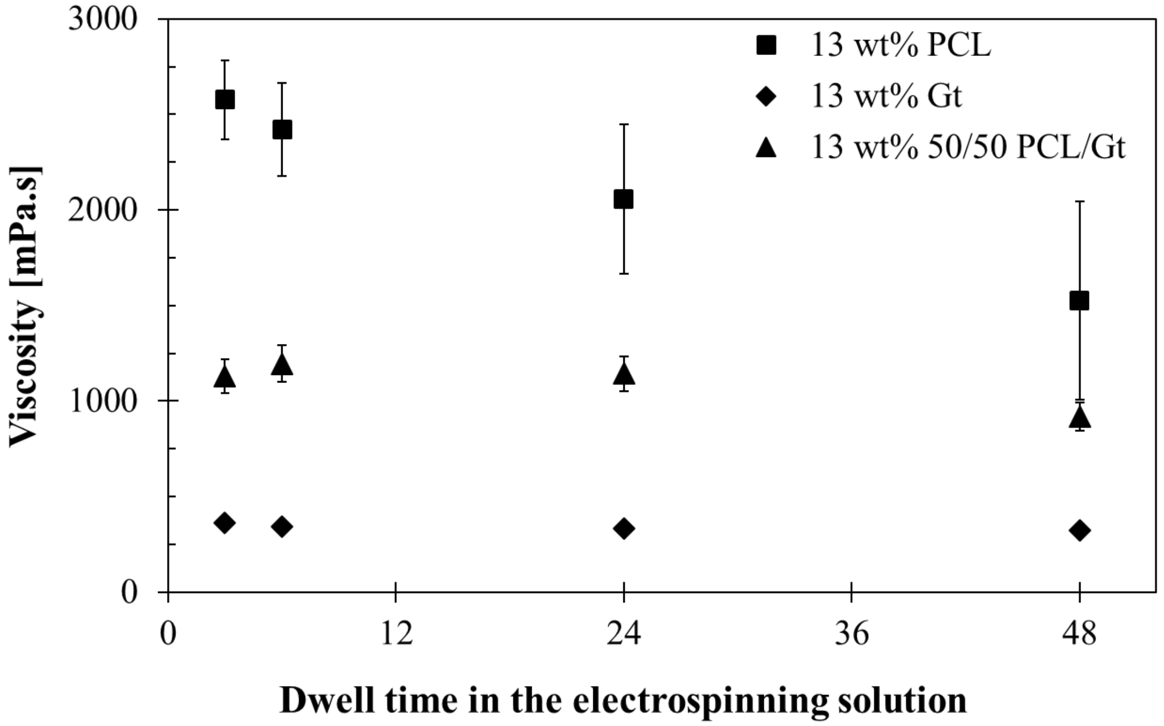

Figure S2. Viscosity measurements of the electrospinning solutions (PCL and/or Gt dissolved in 70/30 AA/FA) with increasing dwell time show that PCL degrades substantially whereas the Gt component remains quite stable.

Characteristic peaks of PCL and Gt in ATR-FTIR

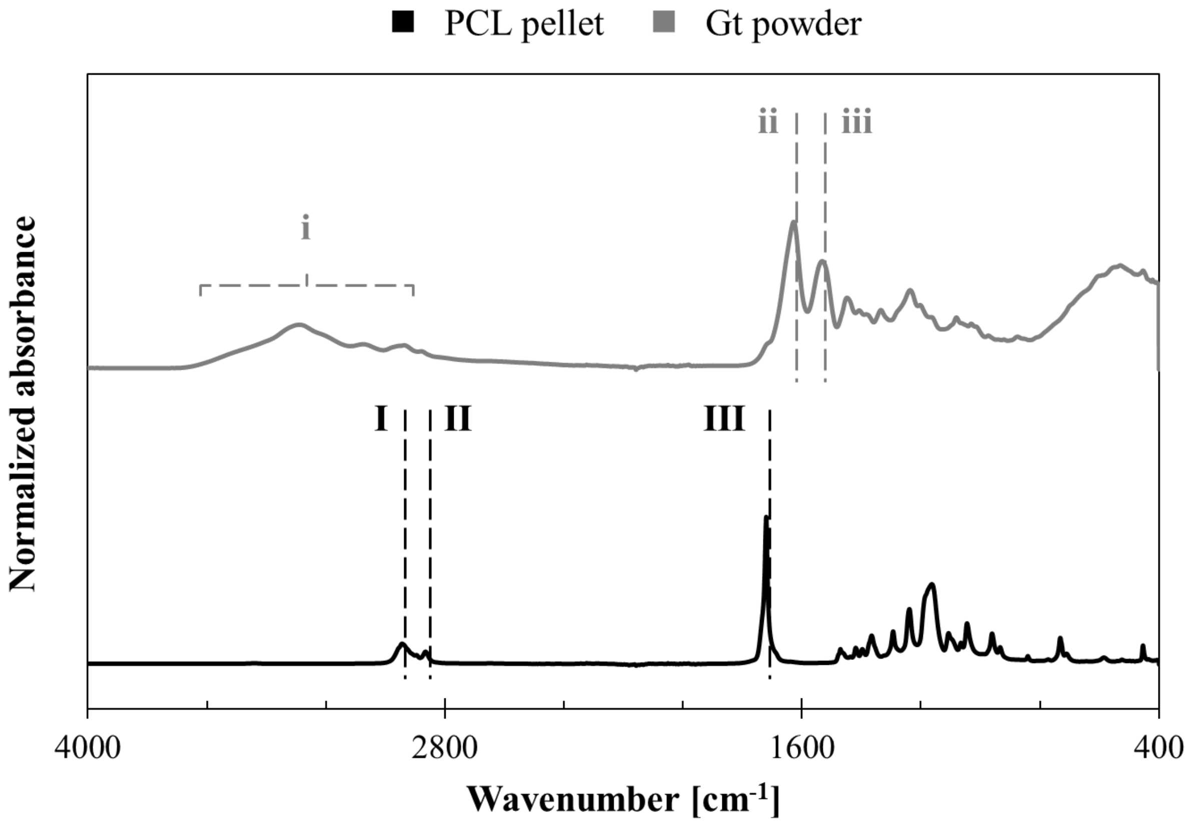

Figure S3. ATR-FTIR spectra of pure PCL and pure Gt, showing their characteristic peaks.

Table S1. Characteristic peaks of a PCL pellet and Gt powder in ATR-FTIR, as indicated in Figure S3.

Peak	Wavenumber (cm^{-1})	Type of vibration
PCL		
I	2936	CH_2 asymmetric stretching
II	2863	CH_2 symmetric stretching
III	1721	C=O stretching
Gt		
i	3100–3500	N-H and O-H stretching (incl. water)
ii	1629	Amide I (C=O stretching)
iii	1525	Amide II (N–H bending)

ATR-FTIR analysis of nanofibers electrospun using different solvent systems

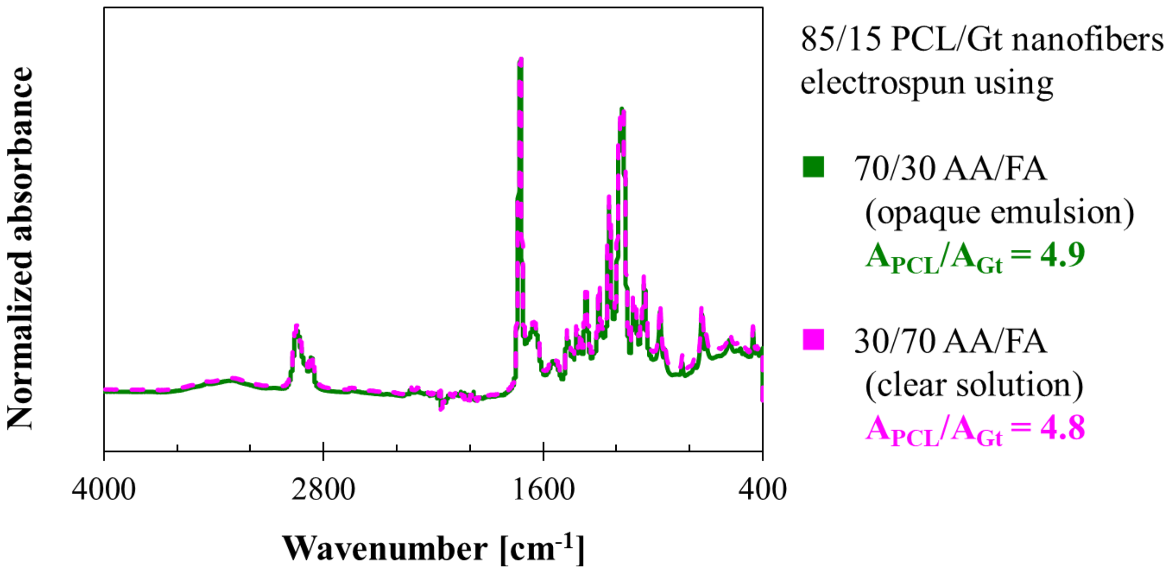

Figure S4. Normalized ATR-FTIR spectra of 85/15 PCL/Gt blend nanofibers electrospun using an emulsion (dissolution in 70/30 AA/FA) or a clear solution (dissolution in 30/70 AA/FA).
